# An Evaluation of a Suicide Prevention E-Learning Program for Police Officers (COPS): Improvement in Knowledge and Competence

**DOI:** 10.3389/fpsyt.2021.770277

**Published:** 2021-12-13

**Authors:** Laura Hofmann, Heide Glaesmer, Marisa Przyrembel, Birgit Wagner

**Affiliations:** ^1^Department of Clinical Psychology and Psychotherapy, Medical School Berlin, Berlin, Germany; ^2^Department of Medical Psychology and Medical Sociology, University of Leipzig, Leipzig, Germany; ^3^Akkon University of Applied Sciences for Human Sciences, Berlin, Germany

**Keywords:** suicide prevention, mental health, police, death notifications, online training (e-learning)

## Abstract

**Background:** Police officers are at high risk for mental and physical health problems and suicidal ideation. The specific risk factors are numerous and concern stressful missions and administrative aspects of the police profession. So far, the police get only little training on specific missions as well as on coping with stress and suicidal ideation in the police profession. In this study we test the efficacy of the online training COPS (Coping with Suicide) for police officers.

**Methods:** A total of 142 police officers from Germany and Switzerland participated in the study; complete data (baseline and post) are available from 102 participants. The training consisted of three modules covering the topics of delivering death notifications, dealing with individuals with suicidal ideation and dealing with one's own distress and suicidal ideation in the police profession. The primary outcomes are perceived knowledge and self-rated competence regarding the contents of the program, actual knowledge as well as symptoms of depression and anxiety (PHQ-9), and attitudes toward suicide (ATTS). The data are collected at baseline and after completing the training.

**Results:** We found a significant increase in knowledge as well as in perceived competence after completing the training. Mental health and attitudes toward suicide did not change significantly. Years on the job had no moderating effect on the effectiveness of the training.

**Discussion:** The results suggest that a short e-learning program significantly improves knowledge and self-rated competence in delivering death notifications, in suicide prevention and stress management. It can be easily integrated into the daily routine of police-officers, and participants could participate at their own pace and from any location. One limitation of this study is the lack of a control-group. Further advantages and limitations of this study are discussed.

**Clinical Trial Registration:** https://www.drks.de/drks_web/, identifier: DRKS00023882.

## Introduction

The police profession is associated with numerous challenges that can have a significant impact on mental and physical health. Several empirical studies show that a high percentage of police officers suffer from mental disorders such as depression, PTSD and substance use disorders (1, 2). In addition, the suicide rate among police officers is higher compared to the general population (3). Physical complaints, such as a high risk of cardiovascular diseases and sleep disorders, are also problems many police officers deal with (4, 5). Such physical and mental health conditions often lead to temporary or permanent periods of inability to work and early retirement (6, 7).

Among the most frequently reported stress factors in the police profession are overwork and shift work, which can have negative effects on the circadian rhythm (4, 8). Further, particular police missions are also repeatedly mentioned as stressors. These include, for example, delivering death notifications, accidents, confrontation with death and the accumulation of these stressful tasks. In addition, there is also the population's increasing tendency toward violence, low social acknowledgment, as well as attacks and insults (1, 9). At the same time, police officers also report that there is often a lack of adequate training for difficult missions, such as delivering death notifications or handling individuals dealing with suicidal behavior. Aftercare is rarely offered after these kinds of potentially traumatic situations, which can also lead to poor mental health. The lack of training opportunities can cause insecurity in the respective situations, and inadequate aftercare causes an increased stress level (10). In parallel, the behavior of the police impacts the well-being of people they deal with during the missions, and inappropriate behavior can lead to the development of psychopathological symptoms in those individuals (11, 12). This also makes it essential to improve the conditions and training opportunities within the police force.

This study focuses on evaluating training to deal with persons with suicidal ideation, to cope with one's own stress and suicidal ideation in the police profession as well as the delivery of death notifications. These topics proved to be the most important and relevant in discussions with the affected group of people.

Police officers have many opportunities for contact with individuals with increased suicide risk: e.g., when people have lost a loved one and become suicidal in response or when the police is called to an individual shortly before a suicide attempt. Therefore, it is highly relevant that police officers should recognize the precursors of suicidal behavior and should be able to assess the severity of suicidal behavior. Most importantly, they should be sufficiently trained in communicating with persons experiencing this specific state in order to be able to react adequately and prevent suicides, if possible.

Although police officers belong to the group of people who frequently come into contact with suicidal behavior, they are not necessarily part of the target group when it comes to gatekeeper training for suicide prevention. This, in turn, often leads to insufficient expertise in this field (10). In addition, studies show that stigmatization and prejudices regarding suicidal behavior and mental disorders are still widespread in the police profession (13, 14). There is now a number of programs with training specifically for police officers in the area of suicide prevention that have shown satisfactory results in the effectiveness of such trainings (10, 15, 16). Those who participated showed a lower stigmatizing attitude toward suicidal behavior, increased knowledge and an increase in self-rated competence in dealing with persons with suicidal ideation. In addition, the individuals with gatekeeper training are more likely to recognize warning signs (16).

These results show that gatekeeper training is quite effective for suicide prevention and that this topic should be more relevant in the police profession.

Police officers are at high risk for mental disorders and suicides. Studies show that the risk of suicide is twice or even three times higher than in the general population in the US (17). As mentioned, police officers are exposed to many distressing factors. In addition, the police profession is still male-dominated, and men generally have a higher risk of suicide than women (18). Moreover, there is easy access to weapons. In their recent systematic review, Syed et al. (2) found a 14.6% prevalence for depression, 14.2% for PTSD, 8.5% for suicidal ideation, and a 25.7% prevalence for hazardous drinking. Stress at the workplace and shift work also have an impact on private life; hence increased social isolation is common, including poor quality relationships with children and partners and also higher divorce rates (19, 20).

One of the major problems among police officers is the low willingness to seek help (21). This is caused by several factors. Mental disorders among the police are still highly stigmatized, and police officers fear bullying among colleagues (22). Absenteeism due to mental health problems is often declared as a weakness, especially among male colleagues. Although specific psychosocial support is already available in many police departments, it is often not used fearing compromised confidentiality (22). Within the police profession, there are generally few opportunities for promotion and career development. For this reason, some worry that getting support may affect this as well. However, external support (e.g., counseling) and internal police support (23) are both avoided. There are often misconceptions about the effects and consequences of psychotherapy (e.g., negative effects on the insurance status).

In sum, there is an urgent need to provide knowledge about support services, to address stigma and to highlight the relevance of mental health. Furthermore, creating low-threshold and possibly anonymous online-based support services is advised in order to offer initial support.

Delivering death notifications is one of the most disliked tasks in the police profession, which many police officers would rather avoid. Delivering a death notification can have a lasting impact not only on the bereaved relatives, but also on the persons delivering the message, such as the police officers (24–26). Studies have shown that in the aftermath the persons delivering the messages often suffer from sadness, insomnia, feelings of helplessness, fear, and guilt (27). Police officers reported great insecurity in dealing with bereaved people, especially when the bereaved people react very emotionally in this situation.

Additionally, police officers are challenged with delivering the message as adequately and empathetically as possible, but at the same time addressing issues relevant to a possible investigation. This often leads to avoidant behavior of the police, which in turn leads to an incorrect delivery of the death notification (12). It is still not uncommon that the message is delivered at the doorstep or even on the phone, or the officers leave the situation as soon as possible after the delivery and do not provide any aftercare for the bereaved (12).

The way a death notification is delivered can impact the mental health and grieving process of the bereaved significantly. An insufficient delivery of the message can clearly lead to PTSD, depression and prolonged grief, as well as to suicidal ideation and increased mortality among the bereaved (11, 12, 28).

Especially when a person has died by suicide, delivering death notifications is even more challenging. Often lacking training on appropriate interaction with this specific group of bereaved leads to growing insecurity in dealing with them (26). Furthermore, one's own attitude as well as personal experience and one's own possible prejudice when delivering the message play an important role. In addition, individuals bereaved by suicide are particularly at risk for mental disorders and increased suicidal ideation (29). This further highlights the relevance of focusing on the way the death notification is delivered to this group.

Despite the far-reaching consequences on both sides in this situation, the people delivering the message repeatedly report lacking education and training in this field (27). In order to best control the negative consequences of this situation for both sides and to keep the process running smoothly, regular training and case work is therefore strongly recommended.

Numerous interventions train in delivering death notifications among different professions (30, 31). Most of these programs have shown greatly increased knowledge; participants feel more competent and better prepared for this situation and report a deeper understanding of the procedure. However, most of these training programs are aimed at doctors, nurses or medical students. To our knowledge, there is little evaluated (online) training specifically aimed at the police.

### Aims and Hypotheses

The main objective of this study is to increase knowledge and self-rated competence regarding suicide prevention. This includes dealing with persons with suicidal ideation, recognizing suicidal tendencies, acting adequately in acute risk situations as well as improving communication skills. Furthermore, the training should improve the ability to deal with one's own mental health distress and possible suicidal ideation. We expected stigmatizing attitudes toward suicidality and symptoms of depression and anxiety to decrease after completing the intervention. In addition, the study aimed to increase knowledge and competence in delivering death notifications. This includes how to deliver messages correctly, how to communicate with the bereaved, and how to support those who are grieving.

Finally, the study aimed to evaluate the roles of age, gender, years in the profession, and knowledge as well as attitudes toward suicide regarding police officer's self-reported competence of suicide prevention, mental health, and delivering death notifications.

## Methods

### Trial Design

This study was a quasi-experimental study. After registration, participants completed a baseline measure and immediately entered the online training and had access to it for three weeks. After completion, participants were asked to take the post-measurement. Three months after the post-measure, participants were sent a follow-up questionnaire.

The study has been approved by the Ethics Committee of the Medical School Berlin (registration number MSB-2020/27) on March 30th, 2020.

### Study Setting and Recruitment

Recruitment took place in Germany and Switzerland. To achieve the largest possible sample, the study cooperated with the police in Berlin, Germany and also with the police in Basel, Switzerland. They integrated the program into their own training courses and supported the data collection by providing computers and working time for the training. Recruitment was also carried out via social networks (e.g., Facebook, Instagram) as well as via police-specific forums and magazines. In addition, recruitment took place via police pastoral care and the police union.

### Eligibility Criteria and Sample Size

The following criteria must be met for participation: (1) working as a police officer, being in training to become a police officer or studying at a police academy, (2) aged between 18 and 67 (retirement age), (3) sufficient German language skills, and (4) having access to the internet. Participants must also submit a signed consent form in order to participate in the training.

There are relatively few studies using online trainings exclusively for the police. We based the sample size and power estimations on results from previous studies examining the effectiveness of online gatekeeper trainings, which focus on topics similar to our training (e.g., suicide prevention) (32). Assuming a between-subject effect size of *d* = 0. 80, power of 0.80, alpha of 0.05 (two sided), and a dropout rate of 40%, the sample size should be at least 100 participants. 142 participants enrolled in the study and 102 participants completed the post measurement.

### Measures

All questionnaires contained measures to assess the following outcomes: demographic variables, an assessment of one's own competence related to the trainings modules, perceived knowledge and competence, attitudes toward suicide and symptoms of depression and anxiety. The post-tests contained additional subjective ratings on the usefulness of the training. The whole questionnaire can be seen in the Supplemental Material.

#### Demographic Variables

We assessed the following parameters for personal information: age, marital status, level of education, year of graduation as a police officer, years of service, current police department and current country they work in. Participants were also asked if they frequently deliver death notifications, how often they did so, if they have been offered any professional support (e.g., psychotherapy) at the moment.

#### Perceived Knowledge and Competence

This questionnaire assesses the participants' perceived knowledge and their own perceived competences on the following subscales: *competence in delivering death notifications* (five items, e.g., “I feel confident in delivering the news of death.”, α = 0.81), *competence in suicide prevention* (seven items, e.g. “When a person expresses suicidal thoughts, I do not know how to proceed.”, α = 0.84), and *competence in managing one's own mental health* (five items, e.g. “I can realistically assess my own psychological stress.”, α = 0.49). The questionnaire consists of 17 items rated on a 6-point Likert scale (“strongly disagree”; “strongly agree”) with higher scores indicating high subjectively perceived competence. A total score can be calculated as well as individual sum scores for each of the three subscales. This questionnaire was designed specifically for this study, and validation is expected.

#### Actual Knowledge

This questionnaire assesses the actual knowledge of the participants based on the training content. The questionnaire consists of 24 items, of which 20 questions have a multiple-choice answer format (“Which group is considered a high-risk group for suicides?”) and four items have an open answer format “Name three risk factors for suicide.”). In the multiple-choice answers, one or more answers may be correct. The higher the total score, the higher the knowledge of the respective person. The participants' answers were evaluated and, if correct, scored with one point per correct answer. This questionnaire was newly developed specifically for this study, as it covered the learning topics of the modules (see Supplementary Material).

#### Short Version of the Patient Health Questionnaire (PHQ-9)

The German version of the PHQ-9 (33) is a screening tool aimed at assessing depression (nine items, 4-point rating scale from “not at all” to “almost every day”), anxiety (five items, yes/no) and functional impairment (one item, 4-point rating scale from “not at all impaired” to “strongly impaired”). The PHQ-9 has shown a good internal consistency of α = 0.89 in a clinical sample (33) and of α = 0.80 in this sample. Higher scores indicate high symptom severity in the respective outcomes.

#### Questionnaire on Attitudes Towards Suicide

The ATTS (Renberg et al., 2003) assesses attitudes toward suicide in the general population as well as the link between those attitudes and suicidal behavior. The questionnaire originally consists of 40 items on a 5-point Likert scale (1 = “strongly agree,” 5 = “strongly disagree”). In this study we adopted the 10-factor model suggested by the authors using 34 items and excluding six items. The internal consistency for the whole instrument was α = 0.60 in a healthy Swedish sample (34) and α = 0.61 in this sample. Six factors showed an internal consistency under α = 0.50 and were excluded from further analyses. The included factors were as follows: suicide as a right (α = 0.76) incomprehensibility (α = 0.53), non-communication (α = 0.55), and normal-common (α = 0.51). In the original study, the internal consistency for the factors varied between α = 0.38 to α = 0.86.

#### Perceived Usefulness of the Training

On the basis of a total of 18 items, the perceived usefulness of the training after completion is assessed. Fifteen of these items are answered on a 6-point Likert scale (“strongly disagree,” “strongly agree”). The questions relate on the one hand to the content of the training (“I now feel safer and more prepared when dealing with individuals with suicidal ideation”) as well as to the design and user-friendliness (“I found the online training to be very well designed.”). The remaining three items are open-ended questions assessing positive as well as negative aspects of the training and suggestions for improvement. The internal consistency for the whole instrument in this study was α = 0.75. This questionnaire (see Supplementary Material) was developed specifically for this study and has not been validated before.

### Procedure

**Figure 1:**
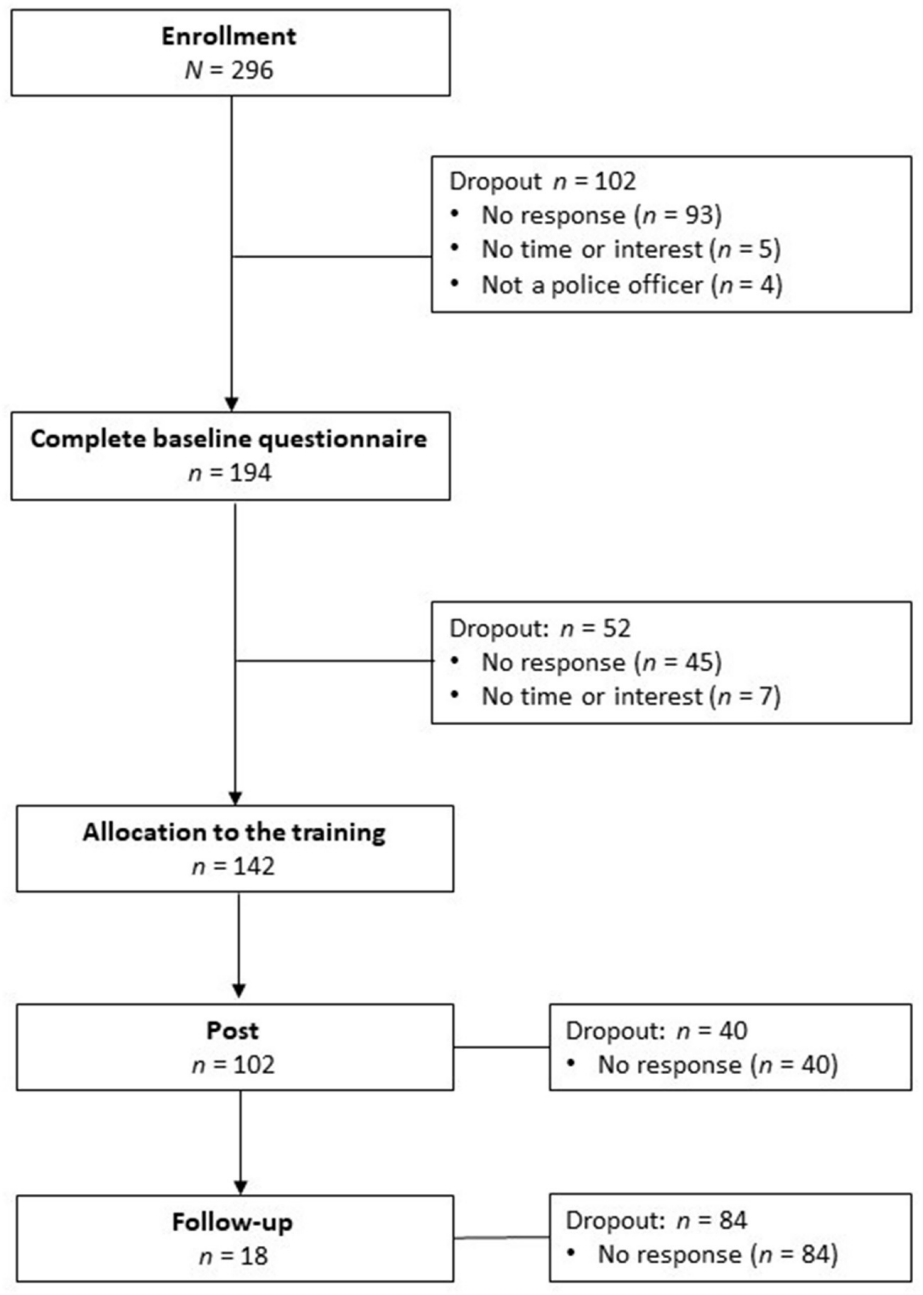
Flowchart of participants.

The flowchart is shown in Figure 1. Interested participants could learn more about the online training on our website (www.cops-praevention.de). The website is still active and is updated regularly. In case of further questions, participants could contact the project coordination at any time. The website has information on the study and on support services, both general and specific for the police.

Participants were requested to register on the website. For this they had to provide their first and last name, a username and e-mail address. They also needed an individual password. After registration, participants received an e-mail with a link to an online survey (baseline) as well as participant information and a consent form for participation. These documents were also provided again as part of the online survey and could be downloaded there. On the first page of the survey, the participants were asked to give their digital consent to participate in the study after reading the participant information. If the participants agreed, they were redirected to the questionnaires. Completing the questionnaire took about 20–25 min and could be interrupted and re-continued or canceled at any time.

After completing the questionnaire, participants were sent technical instructions and the password for using the online training, and they could start the training immediately. Access was valid for three weeks, during which participants should have completed the training. After three weeks the participants were invited to complete the online post-test survey. Once they had completed the questionnaire, participants received an official certificate of participation in the training. Three months after participation, participants were sent a link to the follow-up questionnaire. If participants did not complete the post or follow-up questionnaire, they were reminded again by mail two and four weeks later, respectively.

#### Confidentiality

Participants registered with full name and email address. This information was only accessible to the project coordinator and was needed to assign the participants to the program and to contact them. Logging in to the program required a user name and password. After initial login, participants could reset their username and password. To complete the questionnaires, participants generated a 5-digit code. The collected data were stored under this code. A coding list existed in printed form, which contained the code as well as the name and mail address of the participants and made it possible to assign the codes to the persons. This list was kept in a safe locked cabinet at the Medical School Berlin and was only accessible to the project coordinator. This list was destroyed after the end of the study. Participants were informed that they can request their data be deleted as long as the list exists. After that, it is no longer possible to assign the codes to the names.

### The E-Learning Program

To develop the program, the first author attended police training courses for assessing the needs of police officers. In these seminars the officers were asked what kind of information and content would interest them in a suicide prevention program. Further, they were invited to communicate which situations and circumstances they find most difficult in the police profession. In addition, various professionals were contacted who are familiar with the police (e.g., professors, pastoral care for the police) to include their perspective and their suggestions in the development of the modules. A short online survey was developed to assess the use of support services, aftercare and training opportunities within police stations, and the stress level of certain police operations (*N* = 142) (35). This information was used to develop the three modules of the training. Further, after the e-learning program was developed, a pilot study with students (*N* = 8) of a police academy was conducted and evaluated for its correctness and usefulness. The suggestions and corrections were then incorporated into the training.

#### Modules

**Figure 2:**
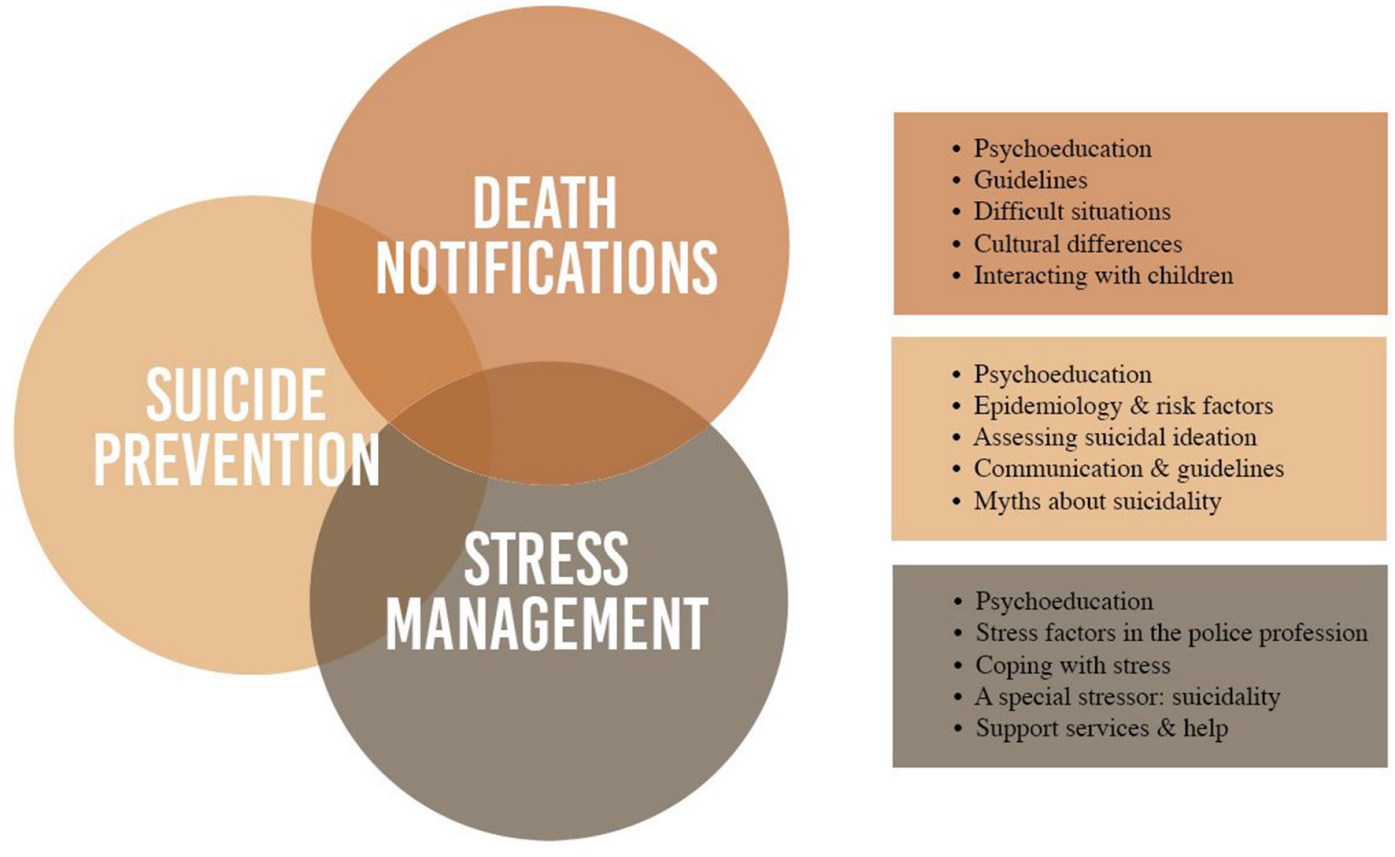
Content of the modules.

The training has three modules: (1) delivering death notifications, (2) dealing with individuals with suicidal ideation and (3) coping with one's own stress and suicidal thoughts in the police profession (see Figure 2 for an illustration of these components and their intersections). The fully detailed contents of each module are in the Supplementary Material.

Working on the tasks of each module takes about 20 min. However, the participants could complete the training independently at their own pace. Participation was free of charge and voluntary. Participants could cancel the training at any time without a reason. Furthermore, the project coordination could be contacted at any time with questions or problems.

The modules are based on the guidelines of the police in Germany for the delivery of death notifications and for dealing with suicidal behavior (36–38). Psychoeducational content is based on the principles of cognitive behavioral therapy.

All modules follow a similar structure: introduction to the topic, psychoeducation, practical guidelines for the police operation as well as advice for communication based on practical case studies. The third module (stress in the police profession) is more practical and also includes methods for recognizing one's own stress, preparing emergency plans in the event of suicidal thoughts, relaxation exercises and information on support services and their consequences for the police profession. Each module contains worksheets for further study of the topic as well as for personal discussion and self-reflection. In addition, there are quizzes with self-grading of the content learned. The training ends with an overview of relevant literature on the various topics.

### The Training Tools

For the training the Wordpress plugin Learnpress was used. This plugin was integrated into an existing Wordpress site and offers the possibility to provide different content for online training. All necessary data protection regulations have been met. The access to the training was protected with a password to prevent access by unauthorized persons. Thus, only those participants can access the training who are allowed to attend the training based on the conditions.

The individual modules are presented as videos with additional audio recordings also with additional worksheets and handouts. To increase knowledge, quizzes are included in the program within each module. The worksheets and handouts could be downloaded by the participants. The training could be completed on the laptop and PC as well as on the smartphone or tablet.

### Statistical Analysis

Data were analyzed using SPSS 25 (39). The demographic data were analyzed using descriptive statistics and frequencies, whereas we presented means, standard deviations, and range for the continuous variables and frequencies for categorical variables. Hierarchical multiple regressions were run to identify factors that influence self-assessed competence at baseline. Paired *t*-tests were used to analyze the change in the respective outcomes from baseline to post. For analyzing any moderating effect of years in the police profession or work location, a moderator analysis was performed using the PROCESS macro for SPSS (40). The questionnaire on the usefulness of the training will be evaluated descriptively to examine how the training was finally evaluated. Cohen's d was used for calculating effect sizes (41). Due to the high amount of missing data at follow-up, these data could not be included in the analysis.

## Results

### Response Rate

A total of *N* = 296 interested individuals registered for the training. Complete baseline data are available from *n* = 194 (65.5% response rate). One hundred and two individuals are considered dropouts, most of whom (*n* = 93) did not report back after registration, *n* = 4 could not be included because they were not police officers, and *n* = 5 lacked time or interest. Of the *n* = 194 participants who were finally enrolled in the study, *n* = 52 did not participate. The reason for this was no time, private problems or the participants did not respond. Complete post-training data are available from *n* = 102 participants (71.8% response rate). We registered a very high attrition rate at follow-up, with only *n* = 18 participants completing the questionnaire (17.6% response rate), therefore, the follow-up data are not included in the analyses. Completers of follow-up did not significantly differ from non-completers regarding sex, age, years in profession, or work place.

### Demographics

More than half of the participants were male (*n* = 63, 61.8%) with an average age of *M* = 38.75 (*SD* = 9.92). 87.3% (*n* = 89) had already completed their police training at the time of participation. Participants had been employed by the police for an average of *M* = 16.14 (*SD* = 10.95) years, and most of the officers were employed by the Swiss police (*n* = 42, 41.2%), followed by participants from the Berlin police (*n* = 38, 37.3%). The remaining participants came from various German states. For further information regarding the participants' demographics, see Table 1. There are different ways to become a police officer in Germany and Switzerland. It is possible to complete a University degree, an apprenticeship or even retraining.

**Table 1:** Characteristics of participants (*N* = 102).

	**M (SD)**	**Range**
Age in years	38.75 (9.92)	19–59
Duration of employment in years	16.14 (10.95)	0.5–43
	**N**	**%**
Gender (male)	63	61.8
Marital status		
Single	18	17.6
In a relationship	19	18.6
Married	50	49.0
Divorced	15	14.7
Education		
Secondary (9 or 10 years of school)	23	22.6
Further (13 years of school)	34	33.3
Higher (university degree)	34	33.3
Apprenticeship (3-year training)	11	10.8
Completed Training as Police Officer	89	87.3
Work location		
Switzerland	42	41.2
Berlin	38	37.3
Other states of Germany	22	21.5

Hierarchical multiple regressions were run to determine if the stepwise addition of demographic factors (age, gender, years in the profession) as well as knowledge and attitudes toward suicide influenced participants' self-assessed competence at baseline. We first analyzed the influence on self-competence in general. The addition of the knowledge-score at baseline led to a statistically significant increase in *R*^2^ of 0.12, *F*_(1, 95)_ = 13.57, *p* < 0.001, indicating that a higher level of knowledge contributed to a higher evaluation of one's own competence. We were able to find the same results for the subscale *competence in delivering death notifications* [*R*^2^= 0.09, *F*_(1, 95)_ = 9.20, *p* = 0.003]. When analyzing the subscale *competence in dealing with people with suicidal ideation*, the addition of knowledge again had a significant influence [*R*^2^ = 0.08, *F*_(1, 95)_ = 9.09, *p* = 0.003], but so did the number of years in the profession, with an increase in *R*^2^ of 0.05, *F*_(1, 97)_ = 5.73, *p* = 0.019, suggesting that the more professional experience a participant has, the more competent they feel in dealing with persons with suicidal tendencies. Last, both knowledge, again [*R*^2^ = 0.08, *F*_(1, 95)_ = 8.92, *p* = 0.004], and also attitudes toward suicide (ATTS) had an impact on how competent participants felt in managing their own stress levels with an increase in *R*^2^ of 0.06, *F*_(1, 97)_ = 6.51, *p* = 0.012, indicating that the more positive attitudes toward suicide and mental health problems are, the more competent participants rate themselves on the subscale *competence in one's own mental health*.

### Program Effectiveness

The results in Table 2 show significant improvement in overall self-assessed competence and in the respective competence subscales. Participants rated themselves as more competent overall after training with 95% CI [−13.89; 8.44], *t*_(101)_ = −8.13, *p* < 0.001. Analyzing the effectiveness of the training concerning the individual subscales, the self-assessed competence regarding the delivery of death notifications (95% CI [−5.59; −3.46], *t*_(101)_ = −8.72, *p* < 0.001), suicide prevention (95% CI [−6.09; −3.26], *t*_(101)_ = −6.56, *p* < 0.001), and dealing with one's own mental health (95% CI [−2.90, −1.12], *t*_(101)_ = −4.48, *p* < 0.001) improved significantly at post-measurement.

**Table 2:** Descriptives and *t*-tests for baseline and post training (*N* = 102).

	**Pre-Training**		**Post-Training**		**t_(101)_**	**95%CI**	***p***	***d***
	***M***	***SD***	***M***	***SD***				
Competence total score	68.63	13.03	79.79	11.13	**−8.13**	**−13.89**, **−8.44**	**<0.001**	0.92
Death Notifications	18.25	5.20	22.74	4.43	**−8.72**	**−5.50**, **−3.46**	**<0.001**	0.93
Suicide Prevention	28.01	6.68	32.69	5.04	**−6.56**	**−6.09**, **−3.26**	**<0.001**	0.79
Mental Health	22.36	3.79	24.37	3.66	**−4.48**	**−2.90**, **−1.12**	**<0.001**	0.54
Knowledge total score	22.44	3.37	26.49	3.90	**−9.41**	**−4.30**, **−4.90**	**<0.001**	1.12
Death Notifications	9.97	1.56	10.97	1.50	**−5.26**	**−1.38**, **−0.62**	**<0.001**	0.65
Suicide Prevention	6.92	1.74	8.45	1.88	**−7.27**	**−1.95**, **−1.11**	**<0.001**	0.85
Mental Health	5.55	1.55	7.07	1.65	**−7.86**	**−1.90**, **−1.14**	**<0.001**	0.95
ATTS total score	2.99	0.26	2.97	0.27	0.83	**–**0.06, 0.06	0.409	0.08
Suicide as a right	2.23	0.61	2.22	0.64	0.22	**–**0.09, 0.11	0.827	0.02
Incomprehensibility	2.89	0.58	2.89	0.63	0.16	**–**0.09, 0.12	0.877	0.00
Non-communication	3.23	0.45	3.25	0.48	**–**0.44	**–**0.11, 0.07	0.664	0.04
Normal-common	2.89	0.68	2.86	0.68	0.38	0.16, 0.37	0.709	0.04
PHQ Depression	3.34	3.33	2.77	2.55	1.86	**–**0.04, 1.18	0.066	0.19
PHQ Anxiety	0.03	0.17	0.01	0.10	1.00	**–**0.02, 0.06	0.320	0.04
PHQ Functional Impairment	0.30	0.52	0.26	0.49	0.815	**–**0.06, 0.13	0.417	0.08

*ATTS, attitudes toward suicide; PHQ, patient health questionnaire*.

*Significant results are in bold*.

Knowledge also improved significantly after completing the training, with 95% CI [−4.30, −4.90], *t*_(101)_ = −9.41, *p* < 0.001. Looking at the individual subscales, knowledge improves significantly regarding delivering death notifications (95% CI [−1.38, −0.62], *t*_(101)_ = −5.26, *p* < 0.001), suicide prevention (95% [CI **–**1.95, −1.11], *t*_(101)_ = −7.27, *p* < 0.001), and mental health (95% [CI **–**1.90, −1.14], *t*_(101)_ = −7.86, *p* < 0.001).

We found almost no significant difference in attitudes toward suicide. In the subscales included, there is almost no change in the mean values after the training.

Furthermore, no improvement could be observed in the participants' symptoms of depression (95% CI [−0.04, 1.18], *t*_(101)_ = 1.66, *p* = 0.066), functional impairment (95% CI [−0.06, 0.13], *t*_(101)_ = 8.15, *p* = 0.417), and anxiety (95% CI [−0.02, 0.06], *t*_(101)_ = 1.00, *p* = 0.320) as a result of the training.

### Moderating Effects

Moderation analyses were run to examine the moderating effect of years in the police profession on changes in self-rated competence and knowledge from baseline to post, since we could find significant changes after completing the training.

Testing for a moderating effect on competence in general, the overall model was significant [*R*^2^ = 0.13, *F*_(3, 98)_ = 4.48, *p* = 0.005], whereas we could not find a significant moderating effect of years in the police profession with Δ*R*^2^ = 0.01, *F*_(1, 98)_ = 0.66, *p* = 0.417, 95% CI [−0.03, 0.01]. The overall model for the subscale *competence in delivering death notifications* was again significant [*R*^2^ = 0.22, *F*_(3, 98)_ = 6.94, *p* < 0.001], but also no significant moderating effect could be found with Δ*R*^2^ = −0.03, *F*_(1, 98)_ = 2.23, *p* = 0.139, 95% CI [−0.03, 0.002]. The overall model for the subscale *competence in suicide prevention* was not significant (*R*^2^ = 0.08, *F*_(3, 98)_ = 2.36, *p* = 0.077), as was the moderating effect with Δ*R*^2^ = 0.003, *F*_(1, 98)_ = 0.19, *p* = 0.667, 95% CI [−0.02, 0.01]. The same results could be found for the subscale *competence in one's own mental health* with *R*^2^ = 0.08, *F*_(3, 98)_ = 1.79, *p* = 0.153 for the overall model and Δ*R*^2^ = 0.01, *F*_(1, 98)_ = 0.42, *p* = 0.516, 95% CI [−0.01, 0.03] for the moderating effect.

We found similar results for the knowledge level after training. Again, the overall model was significant [*R*^2^ = 0.09, *F*_(3, 98)_ = 2.76, *p* = 0.046], but also without any significant moderating effect of years in the profession (Δ*R*^2^ = 0.00, *F*_(1, 98)_ = 0.03, *p* = 0.859, 95% CI [−0.02, 0.02]).

We also tested for a moderating effect of the work location (Germany and Switzerland), but no effect could be found for any outcome (see Supplemental Material).

### Participants' Evaluation of the Program

The majority (*n* = 90, 88.3%) of the participants agreed or strongly agreed that the e-learning program was helpful, 88.7% (*n* = 91) would recommend the training to a colleague, 93.2% (*n* =95) found the content and information well-prepared and presented, and 80.4% (*n* = 82) stated that they had learned important aspects for their work. Three quarters of the participants (*n* = 79, 77.4%) agreed that they feel more secure in delivering death notifications after the completion of the training, and that they could learn new aspects about delivering death notifications (*n* = 86, 84.3%). Regarding dealing with individuals with suicidal ideation, more than three quarters (*n* = 80, 78.4%) somewhat or strongly agreed that they feel more secure after the training, and 76.5% (*n* = 78) stated that they learned more about the topic. 75.5% (*n* = 77) agreed that they would try some suggestions and strategies for dealing with mental stress and that they found it helpful (*n* = 81, 79.4%).

Participants were also able to provide individual feedback, which described the training as a whole as helpful, clearly structured and easily understandable, with a highly relevant selection of topics, and with helpful case studies and suggestions for communication. The additional materials in the form of handouts and worksheets were also mentioned positively. The criticism was mainly related to the fact that the missions in reality usually run differently and there is no fixed procedure to adhere to. Guidelines can therefore only be applied to a limited extent. Some participants found the videos too long, and other participants would have liked more quizzes to test knowledge. Additionally, some reported technical difficulties with some devices.

## Discussion

The aim of this study was to evaluate an online training program for police officers and to improve knowledge and self-rated competence in delivering death notifications, in suicide prevention, and in recognizing one's own stress and suicidality. The e-learning program desired to help police officers better recognize their own stress, offer helpful methods and enhance help-seeking behavior. Furthermore, the program should reduce negative and stigmatizing attitudes about individuals with suicidal ideation and behavior. Along the lines of empirical findings, these three domains represent relevant challenges strongly related to the police profession.

First, we found a significant increase in knowledge after completing the training as well as in self-rated competence. The results are in line with previous studies also finding a significant increase in police officers' knowledge and competence regarding suicidality and depression through gatekeeper training (10, 42).

Increasing knowledge is essential to the profession for many reasons. First, as we found in our sample, higher knowledge leads police officers to rate themselves as more competent. Lacking knowledge is one of the main problems in caring for vulnerable groups (43). With broad knowledge of suicide prevention, police officers may gain confidence in dealing with challenging situations. Even though not all aspects can be addressed by knowledge through training, and complete preparation for missions is not possible, higher self-competence might finally increase self-confidence and decrease stress in such situations. Lack of knowledge and wrong assumptions as well as insecurity due to this are the most common reasons for discrimination and stigmatization (14). Bereaved individuals not infrequently experience stigmatization by the police and therefore perceive contact much more negatively (12). By imparting knowledge, the understanding of persons increased and negative assumptions were reduced, which in turn facilitates better handling of affected persons and also allows police officers to deal with situations more confidently (44).

Further, the role of police officers as gatekeepers in suicide prevention is essential and often underestimated. A competent appearance and knowledge of suicidality can promote adequate interaction with those affected. By informing on suicidality, it can be recognized and assessed more effectively, facilitating identifying risk persons. In their study, Terpstra et al. (45) could show that increased knowledge about suicidality can increase the confidence to talk about it and to help identify individuals at risk.

In the long term, greater knowledge and competence in dealing with one's own stress levels can lead to an increased sensitivity and help better recognize and assess one's own stress. In the police profession, mental disorders are still highly stigmatized. In their study, Karaffa and Koch (21) found that public stigma and self-stigma correlated negatively with seeking help. A broad knowledge of the pathogenesis of stress and mental disorders, as well as education about myths, can help better support those affected and facilitate open communication, since stigma is a barrier to seeking help (44).

Further, we did not find any differences in the other subscales of the ATTS. In their review on gatekeeper training for suicide prevention, Burnette et al. (16) stated that evidence for changing attitudes is limited. One reason for this may be that attitudes do not change within a short period of time. Attitudes may be formed over a longer period of time and be shaped by experiences. Another reason might be that the police-officers' attitudes were already neutral to positive at baseline. Therefore, little positive change is to be expected.

We did not find any significant effect of the training on symptoms of depression and anxiety. There might be a number of reasons for this finding. First, symptoms of depression usually do not improve after such an ultra-short intervention but require a period of time and changes. Second, even though participants were taught coping strategies in module 3, the psycho-educational information about depression was only a small part of the module, with a stronger focus on stress-management.

Finally, it seemed important to analyze a potential moderating effect of years in the police profession. We expected that the longer participants worked as police officers, the more competent they would consider themselves and the more knowledge they would have, meaning these participants would benefit less from the program (10). However, we did not find any moderating effect of years of duty. We also did not find any moderating effect of the country participants work in.

### Limitations

Trainees in this COPS e-learning program could benefit in several ways, as numerous positive outcomes reveal. However, there are limitations of the study. One is that we experienced a high attrition rate at follow up. It already became very difficult to motivate participating police officers to complete the post measure, and the same problem arose during the follow-up. Participants were kindly reminded several times by mail to fill in the questionnaire, but almost none responded. Thus, it is unfortunately not possible to investigate long-term effects of the training. After completing the post questionnaire, participants received an official certificate and were able to take part in a raffle of vouchers. Participants received nothing for the completion of the follow-up questionnaire, which could have significantly reduced their motivation. Interestingly, Arensman et al. (10) as well as Marzano et al. (42) had the same problem with the response rate at follow-up in their trainings for the police. Future studies could address this problem by offering an incentive for completing the follow-up questionnaire. Additional further study information and the importance of the data at the beginning of the program would be useful to improve the overall response rate.

We also had to exclude some subscales due to low internal reliability of the ATTS, which were significant in the pre-post-comparison. The ATTS is used in many international studies (46, 47), but it is also criticized in part because of the generality of the dimensions and the partly low reliabilities (48, 49). In addition, some factors consist of only two or three items, so that internal consistency cannot be improved by excluding the respective items. Consequently, several studies advocate a new factor structure of the ATTS (49).

Furthermore, some of the questionnaires were specially developed for this study and have therefore not yet been validated. Another limitation is the lack of a control group against which we can compare the effects. Further studies should therefore compare one group that undergoes the training as reported here and another group that receives either no training (e.g., as a waitlist control group) or an alternative e-learning program.

## Conclusions

In recent years e-learning programs have proven to be a low threshold alternative to face-to-face training to provide training for different groups, specifically in suicide prevention (32, 50, 51). The increasing digitalization makes online training more and more relevant, becoming an integral part of everyday work life, since it is time-saving and inexpensive. The training could increase knowledge as well as competence in police officers and give police officers the opportunity to educate themselves anonymously and in a low-threshold way.

## Data Availability Statement

The raw data supporting the conclusions of this article will be made available by the authors, without undue reservation.

## Ethics Statement

The studies involving human participants were reviewed and approved by Ethics Committee of the Medical School Berlin, Germany Registration number: MSB-2020/27. The patients/participants provided their written informed consent to participate in this study.

## Author Contributions

LH and BW planned the study, developed the manual and training with the assistance of HG and MP, and wrote the manuscript with the help of HG and MP. LH coordinated the study procedure, programmed the e-learning program, prepared the materials, and performed the analyses. BW supervised the project. All authors contributed to the article and approved the submitted version.

## Conflict of Interest

The authors declare that the research was conducted in the absence of any commercial or financial relationships that could be construed as a potential conflict of interest.

## Publisher's Note

All claims expressed in this article are solely those of the authors and do not necessarily represent those of their affiliated organizations, or those of the publisher, the editors and the reviewers. Any product that may be evaluated in this article, or claim that may be made by its manufacturer, is not guaranteed or endorsed by the publisher.
