# Supplementary material for: An Evaluation of a Suicide Prevention E-Learning Program for Police Officers (COPS): Improvement in Knowledge and Competence
Source: Front Psychiatry. 2021 Dec 13;12:770277. doi: 10.3389/fpsyt.2021.770277 (PMC8710598; doi:10.3389/fpsyt.2021.770277)
Supplement: Supplementary file 1 [file Data_Sheet_1.docx]

**Supplemental Material**

***Questionnaire on perceived knowledge and competence***

|  | Not correct at all |  |  |  |  | Completely correct |
| --- | --- | --- | --- | --- | --- | --- |
| 1. I feel confident in delivering death notifications |  |  |  |  |  |  |
| 1. I don’t know any warning signs of suicide |  |  |  |  |  |  |
| 1. I feel confident in dealing with suicidal individuals |  |  |  |  |  |  |
| 1. I consider my knowledge about suicidality to be good |  |  |  |  |  |  |
| 1. I feel confident in dealing with grieving individuals |  |  |  |  |  |  |
| 1. I’m familiar with the aspects of the process of delivering death notifications |  |  |  |  |  |  |
| 1. If a person expresses suicidal thoughts, I don’t know how to proceed |  |  |  |  |  |  |
| 1. If a person threatens to commit suicide, I know exactly what to do |  |  |  |  |  |  |
| 1. I can realistically assess my own mental stress |  |  |  |  |  |  |
| 1. I don’t know any coping strategies for stress symptoms |  |  |  |  |  |  |
| 1. If I’m not feeling okay, I know where I can get help |  |  |  |  |  |  |
| 1. I’m not informed about the consequences of using psychotherapy |  |  |  |  |  |  |
| 1. I’m well informed about cultural differences in dealing with grief |  |  |  |  |  |  |
| 1. I’m confident in dealing with unexpected reactions of relatives (e.g. laughter) when delivering death notifications |  |  |  |  |  |  |
| 1. I can’t name any risk factors for suicide |  |  |  |  |  |  |
| 1. I can easily assess the severity of suicidal ideation |  |  |  |  |  |  |
| 1. I know how to recognize mental stress in colleagues |  |  |  |  |  |  |

***Questionnaire on actual knowledge***

1. Which term should be used at best?
2. Self-slaughter
3. Self-murder
4. Suicide
5. All terms are fine
6. Name 3 of the most common problems when delivering death notifications.
7. Which of the following terms should be used when delivering death notifications?
8. Technical terms
9. Deceased
10. Dead
11. Corpse
12. What are possible reactions of bereaved individuals?
13. Yelling, severe crying
14. Serenity, rationality
15. Aggressiveness
16. All of the above
17. How much time should you take?
18. As short as possible so that the person can grieve alone
19. Plan enough time to interrogate the persons and clarify organizational questions
20. Plan a lot of time to stay with the person as long as possible
21. Decide individually, depending on the situation
22. If you meet two or more people,
23. It is sufficient if one colleague delivers the message, as less emotional support is needed
24. Can you limit yourself to passing on the information, since the relatives comfort each other and therefore less emotional support is necessary
25. Does it depend on the relationship between the persons whether further support is necessary
26. Should further relatives be notified in any case, so that they can be added as support
27. How should the message be delivered?
28. The relatives should be called beforehand so that they are not frightened when the police are at the door
29. When presenting at the front door, the reason for the appearance should already be mentioned
30. In the home of the relatives, if possible sitting/ at eye level
31. Often it is sufficient to deliver the message and all information directly at the front door
32. When the bereaved ask if they can see the person once again,
33. I advise against it, especially if the corpse is disfigured
34. I leave the decision to the relatives
35. I refer to the mortician
36. I encourage the relatives to do this in any case, as it is important for the processing of grief

9. You have to deliver a death notification and meet a family with children. How do you proceed regarding the children?

1. I leave it up to the parents to decide whether the child should stay
2. The child should be sent to another room
3. Since children usually cannot grasp the situation properly, the child can stay in the room
4. I advise parents not to bring the child with them so that it is not traumatized

10. What do you need to be aware of when you have to deliver a death notification to people of other cultures/religions?

1. In some cultures it is important that only certain persons in the family receive the message (e.g. the men in the family). You should clarify this beforehand.
2. Conflicts can arise regarding autopsy/confiscation of the body, because in some religions the funeral has to take place after a certain time.
3. The mourning and reaction differ only in a few aspects between cultures and religions, so the procedure does not necessarily have to be adapted.
4. It is usually necessary to request reinforcements, as you will encounter extended families in many cultures.

11. How many suicide attempts happen in Germany every year?

1. 80,000
2. 90,000
3. 100,000
4. 110,000

12. Which group is at high risk for suicide?

1. Teenager and young adults (15-25 years)
2. Men over 60 years
3. Women in general
4. Middle-aged women and men (35-60 years)

13. Name 3 risk factors for suicide

14. What are the signs for severe suicidality?

1. Latent planning of suicide
2. Few protective factors
3. Limited self-control
4. All oft the above

15. How should you deal with a person with whom you notice suicidal ideation?

1. I do not address the suidical thoughts in order not to burden the person even more
2. I ask concrete questions to assess the risk
3. I leave the addressing of suicidal ideation to persons who are trained to do so
4. I wait until the person addresses the suicidal thoughts themselves and is willing to talk to me about them

16. What is the best way to talk to a person who wants to commit suicide?

1. Best with 2 or 3 colleagues, so that one can take turns if necessary
2. Preferably alone, so as not to unsettle and frighten the person
3. Preferably together with a relative
4. It is best to talk to the person from a safe distance so that he/she does not feel oppressed

17. What is the best way to approach a person who wants to commit suicide?

1. Get as close as possible in order to be able to hold him/her
2. Let the person determine how far the distance should be
3. Approach the person in such a way that he/she does not notice it if possible and use a moment of surprise in which I can hold and secure the person
4. Do not approach the person at all, but keep a sufficient distance so that the person does not become insecure and thereby commit suicide

18. How do you proceed if you were able to prevent a person from committing suicide or if the person distances himself/herself from the suicidal plan with your help?

1. If the person can assure you that he or she is not suicidal, you can end the assignment, provided that relatives are with the person
2. The person should be taken to the nearest competent psychiatric hospital, even if he/she claims not to be acutely suicidal
3. The person should go to the nearest competent psychiatric hospital, but if he/she is able to make arrangements, he/she can go there alone or with relatives
4. There is no blanket answer, the procedure is decided on a case-by-case basis

19. How many sick days does a police officer have on average per year?

1. 20
2. 35
3. 40
4. 50

20. What can be warning signs for suicidality within yourself?

1. Risk behavior
2. Feeling under pressure all the time
3. Hopelessness
4. Social withdrawal
5. All of the above

21. What is the maximum recommended amount of alcohol for men & women per day?

1. 0.5 liter for both genders
2. 0.4lLiter for women, 0,6 liter for men
3. 0.3 liter for women, 0.5 liter for men
4. 0.4 liter for both genders

22. Name 2 short-term and 2 long-term strategies for coping with stress

23. What is the most important thing when dealing with suicidal ideation?

24. What applies to seeking psychotherapy?

1. The employer knows that you are in treatment because of the financing of the therapy
2. Diagnoses must be sent to the employer on request, so that the further ability to work can be assessed
3. Due to the therapy, your civil servant status may be endangered
4. Therapy is only problematic before you are a registered patient, but not afterwards

***Questionnaire on the usefulness of the training***

|  | Not correct at all |  |  |  |  | Completely correct |
| --- | --- | --- | --- | --- | --- | --- |
| 1. With the help of the training, I was able to learn important aspects that can be useful in my work |  |  |  |  |  |  |
| 1. I don’t have the feeling, that my competences improved through the use of the training |  |  |  |  |  |  |
| 1. I didn’t find the training to be helpful |  |  |  |  |  |  |
| 1. I would recommend the training to my colleagues |  |  |  |  |  |  |
| 1. I found the training to be well designed |  |  |  |  |  |  |
| 1. I found the individual topics insufficiently prepared |  |  |  |  |  |  |
| 1. I now feel more confident and better prepared when delivering death notifications |  |  |  |  |  |  |
| 1. The training reduced my fear of delivering death notifications |  |  |  |  |  |  |
| 1. I could learn certain aspects in delivering death notifications which I want to integrate in my work. |  |  |  |  |  |  |
| 1. I now feel more confident and better prepared when dealing with suicidal individuals |  |  |  |  |  |  |
| 1. The training reduced my fear of dealing with suicidal individuals |  |  |  |  |  |  |
| 1. I could learn certain aspects in dealing with suicidal individuals which I want to integrate in my work. |  |  |  |  |  |  |
| 1. I can well imagine that i will use the suggestions for dealing with stress |  |  |  |  |  |  |
| 1. I did not find the suggestions for stress management and for recognizing psychological distress useful |  |  |  |  |  |  |
| 1. I can imagine that I would now rather use external help if necessary |  |  |  |  |  |  |

What did you find most helpful or positive?

What did you find not helpful or did not like?

Do you have any suggestions for improvement?

*Table 1. Content of the modules*

| **Death notifications** | **Suicide prevention** | **Stress management** |
| --- | --- | --- |
| - Psychoeducation - Grief - Grief after Suicide - Impact of Death Notifications on the bereaved - Guidelines: Delivering death notifications - Preparation - Delivering the notification - Follow-up - FAQs, guidelines for common situations regarding the delivery - Difficult situations and how to deal with them - Cultural differences in grief and dealing with death - Interacting and communicating with children | - Psychoeducation - Epidemiology of Suicide - Male suicide - The role of the police as gatekeeper in suicide prevention - Risk factors - Assessing suicidal ideation - Communication - Getting in contact - Helpful sentences - Sensible conversation - Guidelines: Dealing with individuals shortly before a suicide attempt - Preparation - Communcation - Follow-up - Myths about suicidality | - Psychoeducation - Affective disorders - Substance abuse - Suicadality - Stress factors in the police profession - Police as a high-risk group - Job-related stressors - Coping with stress - Coping strategies - Sleep Hygiene - A special stressor: suicidality - Warning signs - Suicide Safety Plans - Seeking help - Support services and getting help |

*Table 2. Moderator Analysis: Moderating effect of work location (Germany/Switzerland) N = 102*

| Effect | Estimate | *SE* | 95% CI | | *p* |
| --- | --- | --- | --- | --- | --- |
|  |  |  | *LL* | *UL* |  |
| Fixed effects |  |  |  |  |  |
| Intercept | .12 | .19 | -.26 | .50 | .53 |
| Competence | .19 | .26 | -.33 | -.71 | .47 |
| Competence in Delivering | -.02 | .23 | -.48 | .44 | .94 |
| Competence in suicide prevention | .04 | .27 | -.49 | .58 | .87 |
| Competence in mental health | .40 | .33 | -.25 | 1.06 | .22 |
| Knowledge | .45 | .39 | -.33 | 1.22 | .25 |
